# Investigating aquifer contamination and groundwater quality in eastern Terai region of Nepal

**DOI:** 10.1186/s13104-018-3445-z

**Published:** 2018-05-21

**Authors:** Sanjay Mahato, Asmita Mahato, Pankaj Kumar Karna, Nisha Balmiki

**Affiliations:** 1 Aasra Research and Education Academy Counsel, Janapriya Tole, Biratnagar-7, Nepal; 2 Nepal Batawaraniya Sewa Kendra, Biratnagar-9, Nepal

**Keywords:** Physicochemical, Terai, Nepal, Groundwater

## Abstract

**Objective:**

This study aims at assessing the groundwater quality of the three districts of Eastern Terai region of Nepal viz. Morang, Jhapa, Sunsari using physicochemical characteristics and statistical approach so that possible contamination of water reservoir can be understood. pH, temperature, conductivity, turbidity, color, total dissolved solids, fluorides, ammonia, nitrates, chloride, total hardness, calcium hardness, calcium, magnesium, total alkalinity, iron, manganese, arsenic have to be analyzed to know the present status of groundwater quality.

**Results:**

Results revealed that the value of analyzed parameters were within the acceptable limits for drinking water recommended by World Health Organization except for pH, turbidity, ammonia and iron. As per Nepal Drinking Water Quality Standards, fluoride and manganese too were not complying with the permissible limit. Electrical conductivity, total dissolved solids, chloride, total hardness, calcium hardness, manganese, and total alkalinity show good positive correlation with major water quality parameters. Calcium, magnesium, total hardness, calcium hardness and total alkalinity greatly influences total dissolved solids and electrical conductivity. ANOVA, Tukey, and clustering highlight the significance of three districts. Groundwater can be considered safe, but there is always a chance of contamination through chemical wastes in the heavily industrialized area of Morang and Sunsari Industrial corridor.

**Electronic supplementary material:**

The online version of this article (10.1186/s13104-018-3445-z) contains supplementary material, which is available to authorized users.

## Introduction

Groundwater can be defined as water contained in an aquifer matrix located beneath the surface in the saturated zone naturally containing dissolved mineral ions [1–3]. Factors like climate, slope, drainage conditions, water–rock interaction and anthropogenic activities contribute to the groundwater quality [4].

Electrical conductivity is the indicator of dissolved inorganic ions in groundwater. Total dissolved solids describe the inorganic salts and small amounts of organic matter present in water [5]. Low pH of water can cause gastrointestinal disorders [6]. Turbidity in water arises from the presence of very finely divided solids [6]. Dissolved minerals, especially divalent cations cause total hardness in water. Hardness caused by calcium is called calcium hardness, regardless of the salts associated with it [7]. Total alkalinity is the result of the presence of bicarbonates, carbonates and hydroxides of calcium, magnesium and sodium [8].

Excess of fluoride is associated with fluorosis, hyperparathyroidism, increased bone resorption, and skeletal deformity [9–11]. Excess chloride in water is usually taken as an index of pollution and reflected as tracer for groundwater contamination [12, 13]. Nitrate can reach groundwater as a consequence of agricultural activity, wastewater treatment and oxidation of nitrogenous waste products in excreta [14, 15]. Higher contents (up to 3 mg/L) of ammonia are found in strata rich in humic substances or iron or in forests [16].

The high level of manganese causes nervous system disorder, sperm damage, impairments in fertility, nephritis, and nephrolithiasis [17–19]. Arsenic causes cancer [20], hypertension, and cutaneous abnormalities [21].

The objective of this study is to investigate the drinking water quality of groundwater via evaluation of eighteen parameters, determining concentration of contamination if present, comparing values with set standards of World Health Organization (WHO) and Nepal Drinking Water Quality Standards (NDWQS); and finding the correlation among the evaluated parameters.

## Main text

### Methods

#### Study area

**Fig. 1:**
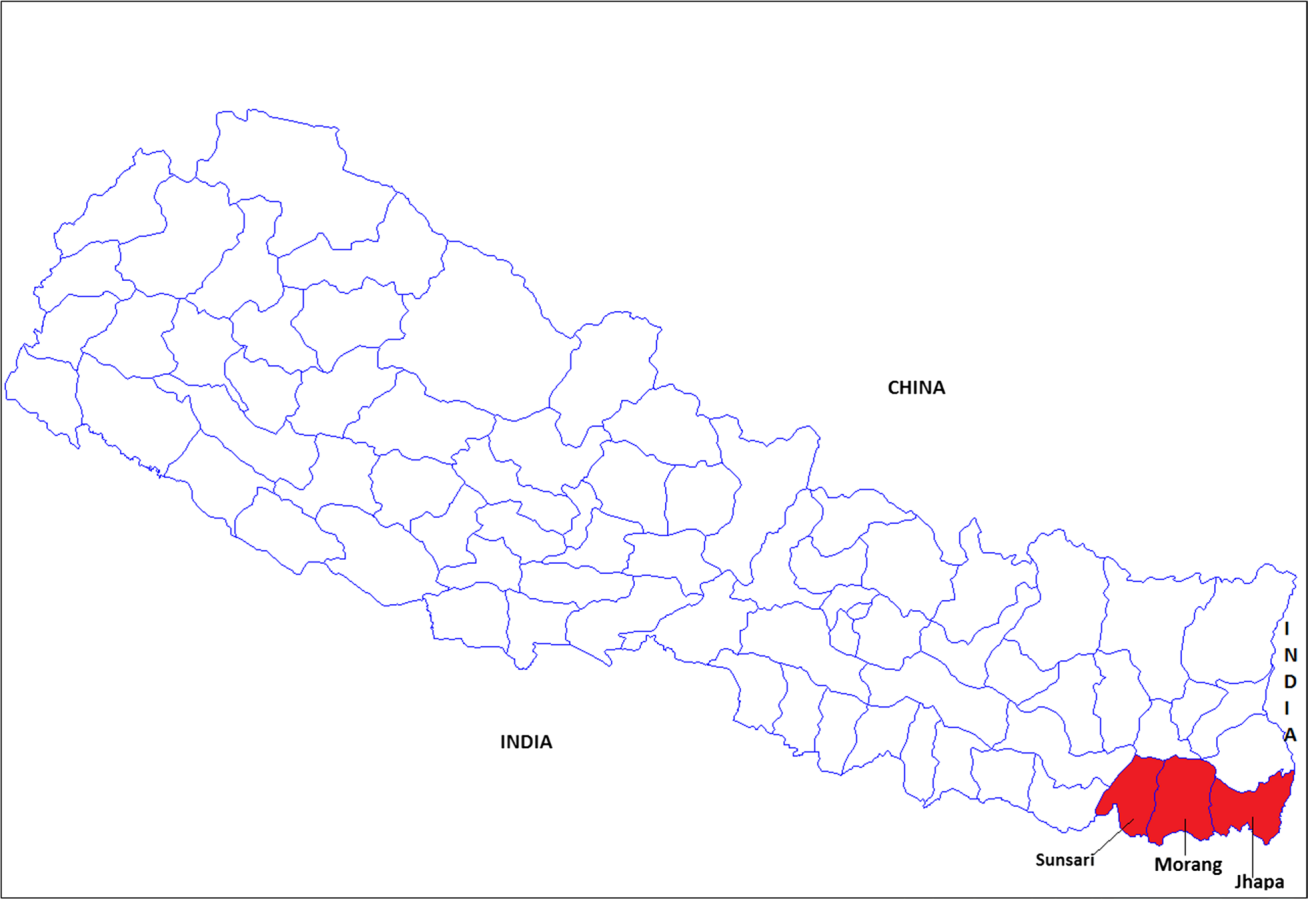
Study area in Eastern Terai of Nepal namely Sunsari, Morang, Jhapa. (Map cited from https://en.wikipedia.org/wiki/List_of_districts_of_Nepal)

The study area is located in the plain area of Eastern Development Region of Nepal comprising of three districts viz. Jhapa (area 1606 km^2^), Morang (area 1855 km^2^) and sunsari (area 1257 km^2^) (Fig. 1). These districts have a tropical climate with annual mean temperature range from 18.8 to 30.1 °C. The annual normal rainfall is 2000–2500 mm [22]. Parts of Sunsari and Morang are a heavily industrialized area due to industrial corridor.

#### Analytical methods

A total of 175 groundwater samples which included 135 borewell and 40 tubewell samples were collected from January, 2015 to September, 2016. Sampling was carried out using pre-cleaned polypropylene bottles. Groundwater was collected in containers after flushing out the tubewells or borewells (minimum 10 min) in order to get the fresh groundwater. Collected samples were preserved at 4 °C and taken into the laboratory for analysis. The parameters were selected on the basis of their relative importance in pollution potential on groundwater. All the physicochemical parameters were determined by the standard methods recommended in American Public Health Association [23].

Physical parameters like temperature, pH, electrical conductivity (EC) and total dissolved solids (TDS) were measured using Thermo Scientific Orion 5-Star Plus instrument. Turbidity (TUR) of the samples were determined by using portable turbidity meter (Hanna instruments HI 93703 Microprocessor).

For chemical parameters such as total alkalinity (TA), total hardness (TH), calcium hardness (CH), major cations such as calcium (Ca^2+^) and magnesium (Mg^2+^), major anion like chlorides (Cl^−^) of groundwater samples were analyzed by titrimetric methods. Nitrates (NO_3_^−^) and fluoride (F^−^) determination in the groundwater samples were carried out by UV Spectrophotometer.

For heavy metal analysis, all the samples were digested with concentrated nitric acid (HNO_3_) to ensure that samples are free of organic impurities. The digested water samples were analyzed for Iron (Fe), Manganese (Mn) and Arsenic (As) using iCE 3000 Series Atomic Absorption Spectrometer (Thermo Scientific) with D2 background correction lamp.

The data were statistically analyzed in October, 2016 using Statistical Package for Social Sciences (SPSS v21) software package. Statistical methods like mean, standard deviation and median for all the parameters were categorically analyzed for borewell, tubewell and total underground water samples. Skewness were normalized by logarithmic and square root approach wherever applicable. Pearson correlation analysis at significance level of 0.05 for all 18 water quality parameters was done to evaluate the degree of interrelationship and association between two variables. As null hypothesis it was hypothesized that there were no significant difference between the given parameter of three districts as a whole or in multiple comparisons. One-way ANOVA was performed along with Tukey’s HSD among all the three districts for all 18 parameters at significance level of 0.05. Hierarchical centroid clustering analysis was performed at significance level of 0.05 using agglomeration schedule and squared euclidean distance interval with all parameters as variables and Label Case by District.

### Results and discussions

Results of the physicochemical characteristics for water samples from 135 borewell and 40 tubewell were analyzed and presented below (Tables 1, 2).

**Table 1:** Statistical summary of the physicochemical parameters of borewell water samples collected from the study area

Parameters	Range	Mean	Standard deviation	Median	WHO guidelines	NDWQS guidelines	Samples not within limit	
							Number	%
Borewell (N = 135)								
pH	5.00–8.36	6.946	0.574	7	6.5–8.5	6.5–8.5	25	18.52
Temp (°C)	17.8–34.8	28.064	4.229	28.7	–	–	–	–
EC (µS/cm)	14.8–1192.0	433.797	233.926	433	–	1500	0	0
Turbidity (NTU)	0.00–93.00	13.508	20.929	3.44	5	5	63	46.67
Color (Hazen)	0.01–4.10	0.235	0.513	0.11	15	5	0	0
TDS (mg/L)	7.50–596.00	223.690	115.611	219.5	1000	1000	0	0
F^−^ (mg/L)	0.02–1.04	0.232	0.193	0.18	1.5	0.5–1.5	0 (119)	0 (88.15)
NH_3_ (mg/L)	0.05–2.53	0.479	0.407	0.38	1.5	1.5	5	3.70
NO_3_^−^ (mg/L)	0.05–2.83	0.187	0.408	0.05	50	50	0	0
Cl^−^ (mg/L)	0.50–145.45	13.424	24.223	3	250	250	0	0
TH (mg/L)	1.11–510.00	190.875	87.955	205.35	–	500	1	0.74
CH (mg/L)	1.00–412.62	119.419	66.504	117.89	–	–	–	–
TA (mg/L)	2.36–512.40	196.491	90.848	209.72	500	–	1	0.74
Fe (mg/L)	0.05–23.10	2.030	3.945	0.59	0.3	0.3	87	64.44
Mn (mg/L)	0.05–3.10	0.356	0.463	0.22	–	0.2	70	51.85
As (mg/L)	0.005–0.050	0.005	0.004	0.005	0.01	0.05	1 (0)	0.74 (0)
Ca^2+^ (mg/L)	0.00–165.05	45.598	26.418	42.708	–	200	0	0
Mg^2+^ (mg/L)	0.00–57.07	18.118	12.255	16.53	–	–	–	–
Tubewell (N = 40)								
pH	5.27–8.59	6.978	0.669	6.965	6.5–8.5	6.5–8.5	9	22.5
Temp (°C)	17.0–35.9	26.563	5.603	28.5	–	–	–	–
EC (µS/cm)	32.2–1898.0	484.998	350.855	461.5	–	1500	(1)	(2.5)
Turbidity (NTU)	0.00–149.00	10.100	24.444	1.915	5	5	15	37.5
Color (Hazen)	0.01–1.03	0.164	0.202	0.125	15	5	0	0
TDS (mg/L)	42.00–949.0	255.820	164.383	235.0	1000	1000	0	0
F^−^ (mg/L)	0.04–1.10	0.245	0.283	0.14	1.5	0.5–1.5	0 (33)	0 (82.5)
NH_3_ (mg/L)	0.05–3.36	0.472	0.545	0.38	1.5	1.5	1	2.5
NO_3_ (mg/L)	0.05–3.38	0.536	0.879	0.11	50	50	0	0
Cl^−^ (mg/L)	0.50–161.80	29.118	40.772	8.0	250	250	0	0
TH (mg/L)	35.00–349.0	194.241	88.628	204.5	–	500	0	0
CH (mg/L)	13.35–270.26	116.957	65.504	107.045	–	–	–	–
TA (mg/L)	36.4–368.88	201.681	87.762	215.67	500	–	0	0
Fe (mg/L)	0.05–10.81	1.184	2.271	0.315	0.3	0.3	20	50
Mn (mg/L)	0.05–2.75	0.402	0.564	0.15	–	0.2	(18)	(45)
As (mg/L)	0.005–0.005	0.005	0.000	0.005	0.01	0.05	0	0
Ca^2+^ (mg/L)	5.34–108.10	43.580	25.797	40.079	–	200	0	0
Mg^2+^ (mg/L)	1.46–61.49	19.889	13.907	15.556	–	–	–	–

Numeric values within bracket represent samples number and percentage of samples not within limit as per NDWQS guidelines while those outside are as per WHO guidelines

*EC* electrical conductivity, *TDS* total dissolved solutes, *TH* total hardness, *CH* calcium hardness, *TA* total alkalinity, *WHO* World Health Organization, *NDWQS* Nepal Drinking Water Quality Standard

**Table 2:** Statistical summary of the physicochemical parameters of groundwater samples collected from the study area

Parameters	Range	Mean	Standard deviation	Median	WHO guidelines	NDWQS guidelines	Samples not within limit	
							Number	%
Groundwater (N = 175)								
pH	5.00–8.59	6.953	0.595	6.99	6.5–8.5	6.5–8.5	34	19.43
Temp (°C)	17.0–35.9	27.721	4.606	28.5	–	–	–	–
EC (µS/cm)	14.8–1898.0	445.500	264.949	436	–	1500	(1)	(0.57)
Turbidity (NTU)	0.00–149.00	12.729	21.756	3.29	5	5	78	44.57
Color (Hazen)	0.01–4.10	0.219	0.461	0.11	15	5	0	0
TDS (mg/L)	7.50–949.00	231.034	128.581	224	1000	1000	0	0
F^−^ (mg/L)	0.02–1.10	0.235	0.216	0.16	1.5	0.5–1.5	0 (152)	0 (86.86)
NH_3_ (mg/L)	0.05–3.36	0.477	0.440	0.38	1.5	1.5	6	3.43
NO_3_ (mg/L)	0.05–3.38	0.266	0.569	0.05	50	50	0	0
Cl^−^ (mg/L)	0.50–161.8	17.011	29.464	3.5	250	250	0	0
TH (mg/L)	1.11–510.00	191.644	87.865	205.35	–	500	(1)	(0.57)
CH (mg/L)	1.00–412.62	118.856	66.097	116.23	–	–	–	–
TA (mg/L)	2.36–512.40	197.678	89.929	213.06	500	–	1	0.57
Fe (mg/L)	0.05–23.10	1.836	3.642	0.46	0.3	0.3	107	61.14
Mn (mg/L)	0.05–3.10	0.367	0.486	0.21	–	0.2	(88)	(50.28)
As (mg/L)	0.005–.050	0.005	0.003	0.005	0.01	0.05	1 (0)	0.57 (0)
Ca^2+^ (mg/L)	0.00–165.05	45.137	26.218	41.281	–	200	0	0
Mg^2+^ (mg/L)	0.00–61.49	18.523	12.632	16.28	–	–	–	–

Numeric values within bracket represent samples number and percentage of samples not within limit as per NDWQS guidelines while those outside are as per WHO

*EC* electrical conductivity, *TDS* total dissolved solutes, *TH* total hardness, *CH* calcium hardness, *TA* total alkalinity, *WHO* World Health Organization, *NDWQS* Nepal Drinking Water Quality Standard

#### Physical characteristics

Out of 175 groundwater samples, 34 (19.43%) samples were out of range from 5.0 to 8.59. pH is mainly influenced by volume of water and soil type. Acidic pH of water may be due to the dissolved carbon dioxide and organic acids from decay and subsequent leaching of plant materials [24]. The range of underground water temperature throughout the seasons were from 17.0 to 35.9 °C. As per NDWQS standard, only one sample showed the result above the permissible limit as 1898 µS/cm. All the samples were found to be within the WHO and NDWQS guideline value. Turbidity in 78 samples (44.57%) were above permissible limit. The origin of turbidity may be clay particles, sewage solids, silt and sand washings, organic and biological sludge and some other factors. Turbidity in water may affect its acceptability to consumers [6]. TDS of all the samples were within the permissible limit of WHO and NDWQS (1000 mg/L).

#### Chemical characteristics

Though WHO has not recommended any guideline, one sample had hardness above NDWQS guidelines. As per classification of hardness [7, 25, 26] only 12% of underground water samples were soft, 9.14% were moderately hard, 14.86% were hard and 64% were very hard. There is no any recommended value for calcium hardness by WHO and NDWQS. Only one sample had alkalinity 512.4 mg/L above WHO guideline. Alkalinity in itself is not harmful to human being, but in large quantity, alkalinity imparts bitter taste to water and may cause eye irritation [6]. Fluoride ion has both beneficial and harmful impact, if not within range. On the basis of WHO, all samples were well within the recommended limit (< 1.5 mg/L). Chloride (0.5–161.8 mg/L) and nitrate concentration (< 0.05–3.38 mg/L) of all the samples were below the permissible limit of WHO and NDWQS. The chloride values in the underground water samples may be due to the dissolution of rocks surrounding the aquifer and probably due to the leakage and anthropogenic pollution like agricultural activities [12, 13]. 6 samples (3.43%) were above the permissible limit for ammonia concentration. Presence of NH_3_ in groundwater indicates influence of industrial effluents and organic contaminants [16].

#### Heavy metals

Among Fe, Mn, and As analysis; only arsenic was below the permissible limits of WHO and NDWQS. 61.14% of samples were above the permissible limit for iron concentration. In 50.28% samples, Mn were found to be above the permissible limit of NDWQS. In the aquifer, groundwater comes in contact with soils, rocks and minerals that naturally contain Fe and Mn and dissolve them, releasing their constituents, including Fe and Mn, to the water [27, 28]. In some local areas high iron content indicates industrial effluent, sewage and landfill leachate [29, 30]. Only one sample showed greater value than WHO guidelines. Although there were no significant signs of leaching in all over studied area, contamination was evident in certain industrial surroundings with iron, steel, textile and paints work [31].

#### Statistical analysis

Analysis of 175 underground water sample (135 borewell water and 40 tubewell sample) indicated that mean and median of most of the parameters were within WHO and NDWQS guidelines except turbidity (mean ± standard deviation 12.73 ± 21.76; median value 3.29), fluoride (0.235 ± 0.216; 0.16), iron (1.836 ± 3.642; 0.46) and manganese (0.367 ± 0.486; 0.21). Fluoride concentration was below the minimum permissible limit of NDWQS, but was within limit of WHO guidelines. The elevated level of iron imparts smell, taste and stain on clothes [32]. Mean and median of Mn were above the permissible limit. High level of Mn causes water discoloration.

Mean value of tubewell water for total hardness and Mg^2+^ was greater than borewell water. It indicates that the tubewell water layer has rocks having minerals rich in magnesium comparative to borewell aquifers surroundings. Dolomite is an ore of Mg. Borewell layer may have minerals like calcite or gypsum in excess. Mean and median value of alkalinity were slightly greater in tubewell water than borewell water. With an overall alkalinity mean of 197.678 ± 89.929 and median of 213.06 for all groundwater samples, it is clear that the pH of water is well buffered. Though mean (0.477 ± 0.44) and median (0.38) value of NH_3_ for all samples were below the permissible limit, its presence in groundwater is not desirable. Mean and median value of nitrate was below the permissible limit 0.266 ± 0.569 and 0.05.

#### Correlations

Electrical conductivity, total dissolved solids, total alkalinity, total hardness, and manganese show good positive correlation with major water quality parameters (Additional file 1: Table S3–S6). Very high correlation between TA and TH (*r *= 0.954), CH and Ca^2+^ (*r *= 0.922), EC and TDS (*r *= 0.898), and TDS and TH (*r* = 0.835) were seen at *p *≤ 0.01. The moderately significant and positive correlation indicated that the presence of calcium, magnesium, total hardness, calcium hardness and total alkalinity greatly influences TDS, TA and EC.

The correlation value suggested that there was a great dependence of total hardness and alkalinity on calcium hardness, calcium and magnesium. Manganese showed low positive correlation (at *p *≤ 0.01) with TH, TA, magnesium, EC, chloride, ammonia, TDS, iron and turbidity. Iron was moderately positive with turbidity and low with ammonia, manganese and color. Though low but positive correlation was shown by ammonia with iron, turbidity, color, manganese, EC, TDS, fluoride and chloride. NH_3_ presence could alter the color of the water. Low positive correlation of chloride on total hardness, total alkalinity, EC, and magnesium was seen. Fluoride showed a poor but positive correlation (at *p *≤ 0.01) with calcium, calcium hardness, color, total alkalinity, EC, ammonia, and TDS. pH was poorly and negatively correlated with turbidity, chloride and iron while low positive correlation was seen with TH, CH, TA, EC, TDS and Ca. Nitrate showed negative and poor correlation with iron and manganese while nitrate was positively correlated with chloride. Temperature, turbidity, fluoride, ammonia and iron were in low positive correlation with color while TH, CH, TA and Mg had poor negative correlation to color.

ANOVA test (Additional file 1: Table S7) showed that pH, EC, color, TDS, chloride, TH, CH, TA, iron, manganese, calcium, and magnesium of three districts were highly significant (*df* = 2, α = 0.05). Tukey’s HSD analysis (Additional file 1: Table S8) presented a district-wise difference, if exists, in parameters (Detailed interpretation in Additional file 1: Table S8). Profile plot of the marginal means of three districts were plotted (Additional file 1: Fig S2–S19). Dendrogram of several parametric combinations (Additional file 1: Fig S20–S24) were plotted showing Hierarchical Cluster Analysis using agglomeration schedule and centroid clustering.

### Conclusion

Undermining pH, turbidity, ammonia, and iron; groundwater in Eastern Terai Districts can be considered safe for drinking and domestic use with certain measures. Iron concentration is a great concern in all these areas. Additionally, the contamination of groundwater with iron and manganese in industrial corridor is high and must be addressed. There is always a chance of groundwater contamination through chemical wastes in the heavily industrialized area of Morang and Sunsari Industrial corridor. It is, however, recommended that a well-designed groundwater monitoring program be devised to periodically screen hazardous contaminants in water.

## Limitations

Soil analysis for fertilizer, pesticides, other chemicals, and extent of leaching wasn’t investigated. Similarly, sewage analysis and analysis of untreated water from industry weren’t performed. Other heavy metals except iron, manganese and arsenic were not evaluated in sample.

## Additional file

**Additional file 1.** Additional Tables and Figures.

## Abbreviations

EC: electrical conductivity
Temp: temperature
Tur: turbidity
TDS: total dissolved solids
F^−^: fluorides
NH_3_: ammonia
NO_3_^−^: nitrates
Cl^−^: chloride
TH: total hardness
CH: calcium hardness
Ca: calcium
Mg: magnesium
TA: total alkalinity
Fe: iron
Mn: manganese
As: arsenic
NDWQS: Nepal Drinking Water Quality Standards
WHO: World Health Organization
