# Supplementary material for: Investigating aquifer contamination and groundwater quality in eastern Terai region of Nepal
Source: BMC Res Notes. 2018 May 21;11:321. doi: 10.1186/s13104-018-3445-z (PMC5963105; doi:10.1186/s13104-018-3445-z)
Supplement: Supplementary file 1 — Additional file 1. Additional Tables and Figures. [file 13104_2018_3445_MOESM1_ESM.docx]

**Table S1** Correlation coefficient of seven parameters of groundwater of Eastern Terai region of Nepal

**Correlations^c^**

|  | | Temp | TH | CH | TA | Ca^2+^ | Mg^2+^ |
| --- | --- | --- | --- | --- | --- | --- | --- |
| pH | Pearson Correlation | -.077 | .283^**^ | .378^**^ | .311^**^ | .357^**^ | .061 |
|  | Sig. (2-tailed) | .312 | .000 | .000 | .000 | .000 | .423 |
| Temp | Pearson Correlation | 1 | -.076 | -.005 | -.103 | -.007 | -.084 |
|  | Sig. (2-tailed) |  | .320 | .951 | .176 | .932 | .266 |
| TH | Pearson Correlation |  | 1 | .777^**^ | .954^**^ | .729^**^ | .658^**^ |
|  | Sig. (2-tailed) |  |  | .000 | .000 | .000 | .000 |
| CH | Pearson Correlation |  |  | 1 | .771^**^ | .922^**^ | .162^*^ |
|  | Sig. (2-tailed) |  |  |  | .000 | .000 | .032 |
| TA | Pearson Correlation |  |  |  | 1 | .762^**^ | .622^**^ |
|  | Sig. (2-tailed) |  |  |  |  | .000 | .000 |
| Ca^2+^ | Pearson Correlation |  |  |  |  | 1 | .062 |
|  | Sig. (2-tailed) |  |  |  |  |  | .414 |

**. Correlation is significant at the 0.01 level (2-tailed).

*. Correlation is significant at the 0.05 level (2-tailed).

| c. Listwise N=175  Pearson correlation analysis (2-tailed) at significance level of 0.05 and 0.01 for water quality parameters like pH, Temperature (Temp.), TH- Total Hardness, CH- Calcium Hardness, TA-Total Alkalinity, and Calcium (Ca^2+^) in order to evaluate the degree of interrelationship and association between two variables. |
| --- |

**Table S2** Correlation coefficient of seven parameters of groundwater of Eastern Terai region of Nepal

**Correlations^c^**

|  | | Cl^-^ | EC | TDS | pH | Temp | TH | CH | TA | Ca^2+^ | Mg^2+^ |
| --- | --- | --- | --- | --- | --- | --- | --- | --- | --- | --- | --- |
| Tur | Pearson Correlation | .146 | .013 | .055 | -.197^**^ | .050 | .021 | -.005 | .043 | -.006 | .012 |
|  | Sig. (2-tailed) | .053 | .865 | .466 | .009 | .508 | .787 | .945 | .569 | .937 | .872 |
| Cl^-^ | Pearson Correlation | 1 | .351^**^ | .424^**^ | -.289^**^ | .029 | .228^**^ | .060 | .210^**^ | .071 | .256^**^ |
|  | Sig. (2-tailed) |  | .000 | .000 | .000 | .699 | .002 | .431 | .005 | .350 | .001 |
| EC | Pearson Correlation |  | 1 | .898^**^ | .156^*^ | .026 | .760^**^ | .527^**^ | .730^**^ | .488^**^ | .573^**^ |
|  | Sig. (2-tailed) |  |  | .000 | .040 | .736 | .000 | .000 | .000 | .000 | .000 |
| TDS | Pearson Correlation |  |  | 1 | .172^*^ | .064 | .835^**^ | .642^**^ | .798^**^ | .607^**^ | .566^**^ |
|  | Sig. (2-tailed) |  |  |  | .023 | .403 | .000 | .000 | .000 | .000 | .000 |

**. Correlation is significant at the 0.01 level (2-tailed).

*. Correlation is significant at the 0.05 level (2-tailed).

c. Listwise N=175

Pearson correlation analysis (2-tailed) at significance level of 0.05 and 0.01 for water quality parameters like Turbidity (Tur), Chloride (Cl^-^), Electrical Conductivity (EC), and Total Dissolved Solutes (TDS) against Turbidity (Tur), Chloride (Cl^-^), Electrical Conductivity (EC), and Total Dissolved Solutes (TDS), pH, Temperature (Temp.), TH- Total Hardness, CH- Calcium Hardness, TA-Total Alkalinity, Calcium (Ca^2+^), and Magnesium (Mg^2+^) in order to evaluate the degree of interrelationship and association between two variables.

**Table S3** Correlation coefficient of seven parameters of groundwater of Eastern Terai region of Nepal

**Correlations^c^**

|  | | F^-^ | NH_3_ | NO_3_^-^ | Fe | Mn | As |
| --- | --- | --- | --- | --- | --- | --- | --- |
| Color | Pearson Correlation | .238^**^ | .379^**^ | -.114 | .268^**^ | .027 | -.055 |
|  | Sig. (2-tailed) | .002 | .000 | .132 | .000 | .726 | .467 |
| F^-^ | Pearson Correlation | 1 | .162^*^ | -.048 | .031 | -.035 | -.085 |
|  | Sig. (2-tailed) |  | .032 | .525 | .680 | .647 | .264 |
| NH_3_ | Pearson Correlation |  | 1 | -.092 | .418^**^ | .233^**^ | -.117 |
|  | Sig. (2-tailed) |  |  | .228 | .000 | .002 | .124 |
| NO^3-^ | Pearson Correlation |  |  | 1 | -.152^*^ | -.151^*^ | -.038 |
|  | Sig. (2-tailed) |  |  |  | .044 | .046 | .621 |
| Fe | Pearson Correlation |  |  |  | 1 | .399^**^ | -.070 |
|  | Sig. (2-tailed) |  |  |  |  | .000 | .354 |
| Mn | Pearson Correlation |  |  |  |  | 1 | -.034 |
|  | Sig. (2-tailed) |  |  |  |  |  | .652 |
| As | Pearson Correlation |  |  |  |  |  | 1 |
|  | Sig. (2-tailed) |  |  |  |  |  |  |

**. Correlation is significant at the 0.01 level (2-tailed).

*. Correlation is significant at the 0.05 level (2-tailed).

c. Listwise N=175

Pearson correlation analysis (2-tailed) at significance level of 0.05 and 0.01 for water quality parameters like Color, Fluoride (F^-^), Ammonia (NH_3_), Nitrate (NO^3-^), Iron (Fe), Manganese (Mn), and Arsenic (As) in order to evaluate the degree of interrelationship and association between two variables.

**Table S4** Correlation coefficient of several parameters of groundwater of Eastern Terai region of Nepal

|  | | Color | F | NH_3_ | NO_3_^-^ | Fe | Mn | As |
| --- | --- | --- | --- | --- | --- | --- | --- | --- |
| pH | Pearson Correlation | -.075 | .148 | .004 | -.109 | -.265^**^ | -.107 | .015 |
|  | Sig. (2-tailed) | .325 | .051 | .956 | .151 | .000 | .158 | .847 |
| Temp | Pearson Correlation | .251^**^ | .135 | .138 | -.043 | -.060 | -.112 | -.126 |
|  | Sig. (2-tailed) | .001 | .074 | .069 | .573 | .428 | .141 | .098 |
| TH | Pearson Correlation | -.254^**^ | .116 | .133 | -.064 | -.063 | .257^**^ | -.109 |
|  | Sig. (2-tailed) | .001 | .128 | .080 | .400 | .409 | .001 | .150 |
| CH | Pearson Correlation | -.151^*^ | .277^**^ | .057 | -.123 | -.045 | .086 | -.111 |
|  | Sig. (2-tailed) | .045 | .000 | .453 | .104 | .558 | .258 | .143 |
| TA | Pearson Correlation | -.250^**^ | .187^*^ | .114 | -.053 | -.030 | .254^**^ | -.106 |
|  | Sig. (2-tailed) | .001 | .013 | .133 | .487 | .698 | .001 | .164 |
| Ca^2+^ | Pearson Correlation | -.123 | .305^**^ | .038 | -.095 | -.087 | .071 | -.103 |
|  | Sig. (2-tailed) | .104 | .000 | .616 | .210 | .252 | .347 | .176 |
| Mg^2+^ | Pearson Correlation | -.219^**^ | -.097 | .071 | .018 | -.049 | .241^**^ | .240^**^ |
|  | Sig. (2-tailed) | .004 | .201 | .354 | .813 | .516 | .001 | .001 |
| Tur | Pearson Correlation | .329^**^ | .107 | .394^**^ | -.103 | .587^**^ | .262^**^ | -.055 |
|  | Sig. (2-tailed) | .000 | .159 | .000 | .174 | .000 | .000 | .467 |
| Cl | Pearson Correlation | .065 | .146 | .157^*^ | .249^**^ | .148 | .243^**^ | -.071 |
|  | Sig. (2-tailed) | .391 | .055 | .038 | .001 | .051 | .001 | .352 |
| EC | Pearson Correlation | -.140 | .179^*^ | .223^**^ | .055 | .002 | .227^**^ | -.108 |
|  | Sig. (2-tailed) | .064 | .018 | .003 | .467 | .976 | .002 | .155 |
| TDS | Pearson Correlation | -.134 | .153^*^ | .205^**^ | .030 | .006 | .256^**^ | -.124 |
|  | Sig. (2-tailed) | .077 | .043 | .007 | .693 | .933 | .001 | .102 |

**. Correlation is significant at the 0.01 level (2-tailed).

*. Correlation is significant at the 0.05 level (2-tailed).

Pearson correlation analysis (2-tailed) at significance level of 0.05 and 0.01 for water quality parameters like pH, Temperature (Temp.), TH- Total Hardness, CH- Calcium Hardness, TA-Total Alkalinity, Calcium (Ca^2+^), Magnesium (Mg^2+^), Turbidity (Tur), Chloride (Cl^-^), Electrical Conductivity (EC), and Total Dissolved Solutes (TDS) against Color, Fluoride (F^-^), Ammonia (NH_3_), Nitrate (NO_3_^-^), Iron (Fe), Manganese (Mn), and Arsenic (As), in order to evaluate the degree of interrelationship and association between two variables.

**Table S5** ANOVA Test Result of all the parameters among three districts viz. Morang, Sunsari and Jhapa

| **Parameters** | **F score** | **Significance Level** | **Inference** |
| --- | --- | --- | --- |
| pH | 12.214 | 0.000 | Highly significant i.e. mean pH among the district is statistically different from each other. |
| Temperature | 2.848 | 0.061 | Non-significant i.e. mean temperature among the district is statistically similar. |
| Electrical Conductivity (EC) | 15.681 | 0.000 | Highly significant i.e. mean Electrical Conductivity among the district is statistically different from each other. |
| Turbidity | 2.689 | 0.071 | Non-significant i.e. mean turbidity among the district is statistically similar. |
| Color | 14.195 | 0.000 | Highly significant i.e. mean color among the district is statistically different from each other. |
| Total Dissolved Solutes (TDS) | 21.975 | 0.000 | Highly significant i.e. mean TDS among the district is statistically different from each other. |
| F^-^ | 1.898 | 0.153 | Non-significant i.e. mean fluoride (F^-^) among the district is statistically similar. |
| NH_3_ | 1.649 | 0.195 | Non-significant i.e. mean ammonia (NH_3_) among the district is statistically different from each other. |
| NO_3_^-^ | 0.038 | 0.963 | Non-significant i.e. mean nitrate (NO_3_^-^) among the district is statistically similar. |
| Cl^-^ | 11.995 | 0.000 | Highly significant i.e. mean chloride (Cl^-^) among the district is statistically different from each other. |
| Total Hardness (TH) | 27.230 | 0.000 | Highly significant i.e. mean total hardness (TH) among the district is statistically different from each other. |
| Calcium Hardness (CH) | 10.477 | 0.000 | Highly significant i.e. mean Calcium Hardness (CH) among the district is statistically different from each other. |
| Total Alkalinity (TA) | 26.127 | 0.000 | Highly significant i.e. mean Total Alkalinity (TA) among the district is statistically different from each other. |
| Fe | 3.837 | 0.023 | Highly significant i.e. mean iron (Fe) among the district is statistically different from each other. |
| Mn | 4.090 | 0.018 | Highly significant i.e. mean manganese (Mn) among the district is statistically different from each other. |
| As | 0.806 | 0.448 | Non-significant i.e. mean Arsenic (As) among the district is statistically similar. |
| Ca^2+^ | 7.847 | 0.001 | Highly significant i.e. mean calcium ion (Ca^2+^) among the district is statistically different from each other. |
| Mg^2+^ | 21.468 | 0.000 | Highly significant i.e. mean magnesium ion (Mg^2+^) among the district is statistically different from each other. |

| **Table S6** Multiple Comparisons (Tukey’s HSD Test) to identify where the differences lie | | | | | | | | |
| --- | --- | --- | --- | --- | --- | --- | --- | --- |
| Dependent  Variable | | (i) District | (j) District | Mean Difference  (i-j) | Std. Error | Sig. | 95% Confidence Interval | |
|  |  |  |  |  |  |  | Lower Bound | Upper Bound |
| pH | Tukey HSD | J | M | -.5217^*^ | .11931 | .000 | -.8037 | -.2396 |
|  |  | J | S | -.6079^*^ | .12897 | .000 | -.9128 | -.3030 |
|  |  | M | S | -.0862 | .09604 | .642 | -.3133 | .1408 |
| Temp | Tukey HSD | J | M | 1.245 | .9706 | .407 | -1.049 | 3.540 |
|  |  | J | S | 2.448 | 1.0492 | .054 | -.033 | 4.928 |
|  |  | M | S | 1.203 | .7813 | .275 | -.645 | 3.050 |
| TH | Tukey HSD | J | M | -117.3758^*^ | 16.40192 | .000 | -156.1529 | -78.5987 |
|  |  | J | S | -111.9629^*^ | 17.73120 | .000 | -153.8827 | -70.0432 |
|  |  | M | S | 5.4129 | 13.20324 | .912 | -25.8020 | 36.6277 |
| CH | Tukey HSD | J | M | -54.6323^*^ | 13.36686 | .000 | -86.2339 | -23.0306 |
|  |  | J | S | -62.7111^*^ | 14.45017 | .000 | -96.8739 | -28.5483 |
|  |  | M | S | -8.0788 | 10.76008 | .734 | -33.5176 | 17.3599 |
| TA | Tukey HSD | J | M | -118.9342^*^ | 16.86947 | .000 | -158.8167 | -79.0518 |
|  |  | J | S | -111.1252^*^ | 18.23664 | .000 | -154.2399 | -68.0104 |
|  |  | M | S | 7.8091 | 13.57961 | .834 | -24.2956 | 39.9137 |
| Ca^2+^ | Tukey HSD | J | M | -18.7934^*^ | 5.37583 | .002 | -31.5028 | -6.0839 |
|  |  | J | S | -21.9866^*^ | 5.81151 | .001 | -35.7261 | -8.2472 |
|  |  | M | S | -3.1933 | 4.32744 | .741 | -13.4241 | 7.0376 |
| Mg^2+^ | Tukey HSD | J | M | -15.7988^*^ | 2.42036 | .000 | -21.5210 | -10.0767 |
|  |  | J | S | -10.9478^*^ | 2.61651 | .000 | -17.1337 | -4.7619 |
|  |  | M | S | 4.8511^*^ | 1.94834 | .036 | .2448 | 9.4573 |
| Tur | Tukey HSD | J | M | .2921 | .12866 | .063 | -.0121 | .5962 |
|  |  | J | S | .2690 | .13909 | .132 | -.0598 | .5978 |
|  |  | M | S | -.0231 | .10357 | .973 | -.2679 | .2218 |
| Cl^-^ | Tukey HSD | J | M | -.1787 | .11957 | .296 | -.4614 | .1040 |
|  |  | J | S | .2928 | .12926 | .064 | -.0128 | .5984 |
|  |  | M | S | .4714^*^ | .09625 | .000 | .2439 | .6990 |
| EC | Tukey HSD | J | M | -7.1288^*^ | 1.27318 | .000 | -10.1389 | -4.1188 |
|  |  | J | S | -5.3199^*^ | 1.37637 | .000 | -8.5739 | -2.0659 |
|  |  | M | S | 1.8089 | 1.02489 | .185 | -.6141 | 4.2320 |
| TDS | Tukey HSD | J | M | -5.3908^*^ | .81808 | .000 | -7.3249 | -3.4567 |
|  |  | J | S | -4.5555^*^ | .88438 | .000 | -6.6463 | -2.4647 |
|  |  | M | S | .8353 | .65854 | .415 | -.7216 | 2.3922 |
| Color | Tukey HSD | J | M | .2795^*^ | .05661 | .000 | .1457 | .4134 |
|  |  | J | S | .2996^*^ | .06120 | .000 | .1549 | .4443 |
|  |  | M | S | .0201 | .04557 | .898 | -.0876 | .1278 |
| F^-^ | Tukey HSD | J | M | .0195 | .04107 | .883 | -.0776 | .1166 |
|  |  | J | S | .0745 | .04440 | .217 | -.0305 | .1795 |
|  |  | M | S | .0549 | .03306 | .223 | -.0232 | .1331 |
| NH_3_ | Tukey HSD | J | M | .1034 | .05700 | .168 | -.0313 | .2382 |
|  |  | J | S | .0815 | .06162 | .385 | -.0642 | .2271 |
|  |  | M | S | -.0220 | .04588 | .881 | -.1305 | .0865 |
| NO_3_^-^ | Tukey HSD | J | M | .0197 | .07191 | .959 | -.1503 | .1897 |
|  |  | J | S | .0142 | .07774 | .982 | -.1696 | .1980 |
|  |  | M | S | -.0055 | .05789 | .995 | -.1423 | .1314 |
| Fe | Tukey HSD | J | M | .4988^*^ | .19016 | .026 | .0492 | .9483 |
|  |  | J | S | .2489 | .20557 | .448 | -.2371 | .7349 |
|  |  | M | S | -.2498 | .15308 | .235 | -.6117 | .1121 |
| Mn | Tukey HSD | J | M | -.1664^*^ | .06487 | .030 | -.3198 | -.0130 |
|  |  | J | S | -.0634 | .07013 | .638 | -.2292 | .1024 |
|  |  | M | S | .1030 | .05222 | .122 | -.0205 | .2264 |
| As | Tukey HSD | J | M | -.0023 | .00255 | .640 | -.0083 | .0037 |
|  |  | J | S | .0000 | .00275 | 1.000 | -.0065 | .0065 |
|  |  | M | S | .0023 | .00205 | .502 | -.0025 | .0071 |

Based on observed means.

The error term is Mean Square (Error) = .000.

*. The mean difference is significant at the .05 level.

Temperature (Temp), Turbidity (Tur), fluoride (F), ammonia (NH3), nitrate (NO3-), and arsenic (As) of the groundwater of all the three districts namely Jhapa, Morang and Sunsari district were non-significant i.e. similar among themselves. Magnesium (Mg) of the groundwater of all the three districts namely Jhapa (J), Morang (M) and Sunsari (S) district were significantly different among themselves.

pH, total hardness (TH), calcium hardness (CH), total alkalinity (TA), calcium (Ca), electrical conductivity (EC), total dissolved solutes (TDS), and color of the groundwater of Jhapa (J) district were significantly different with Morang (M) and Sunsari (S) while Morang and Sunsari showed no significant difference in these parameters between them.

Iron (Fe) and manganese (Mn) of the groundwater of Jhapa and Morang were significantly different from one another while Sunsari was non-significant with Morang and Jhapa. Chloride (Cl) of the groundwater of Morang and Sunsari were significantly different from one another while Jhapa was non-significant with Morang and Sunsari.


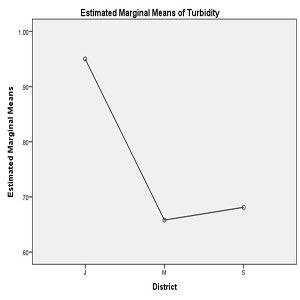

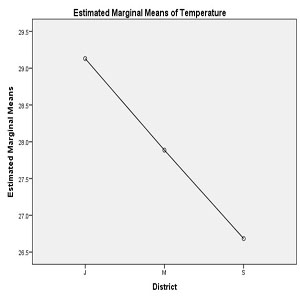


Fig S1 Fig S2


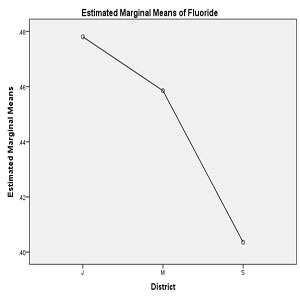

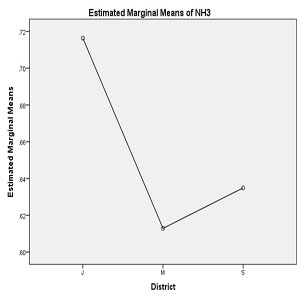


Fig S3 Fig S4


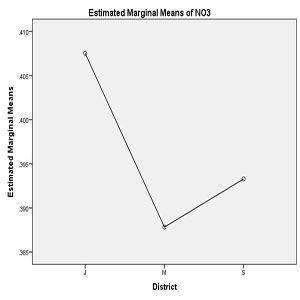

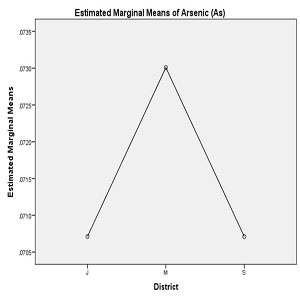


Fig S5 Fig S6


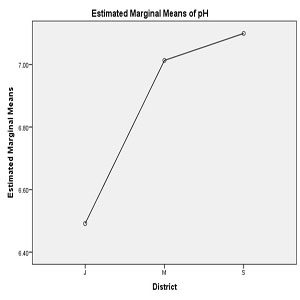

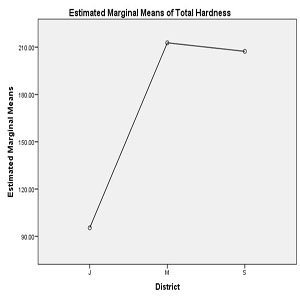


Fig S7 Fig S8


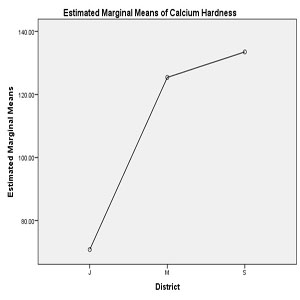

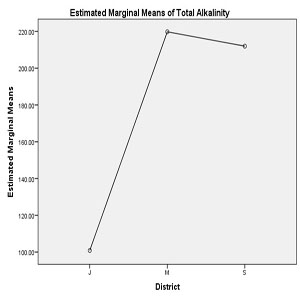


Fig S9 Fig S10


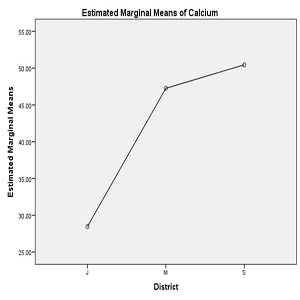

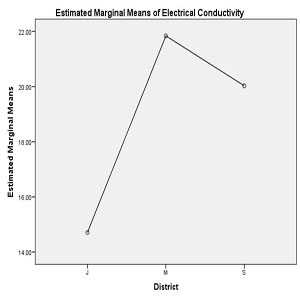


Fig S11 Fig S12


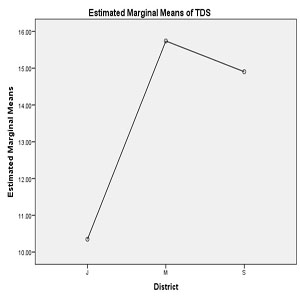

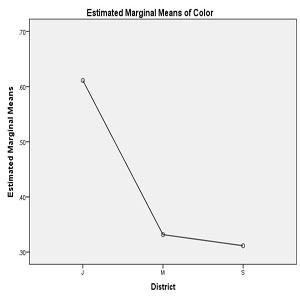


Fig S13 Fig S14


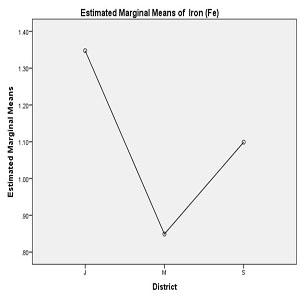

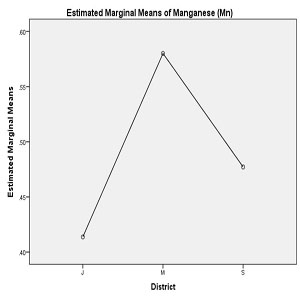


Fig S15 Fig S16


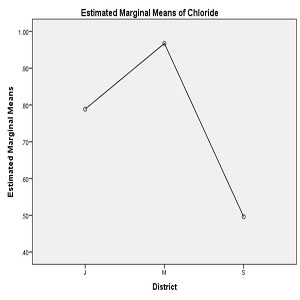

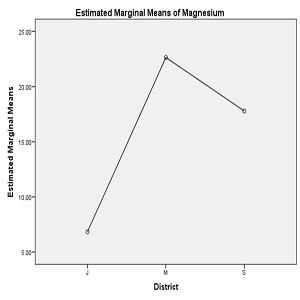


Fig S17 Fig S18

Fig S1 to Fig S18 shows the profile plot of the marginal means of three districts namely Jhapa (symbolized as J), Morang (symbolized as M), and Sunsari (symbolized as S) for each parameter.


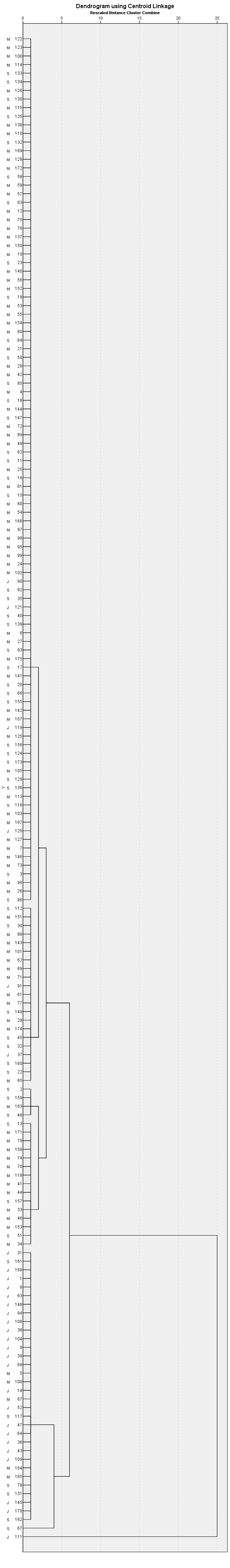

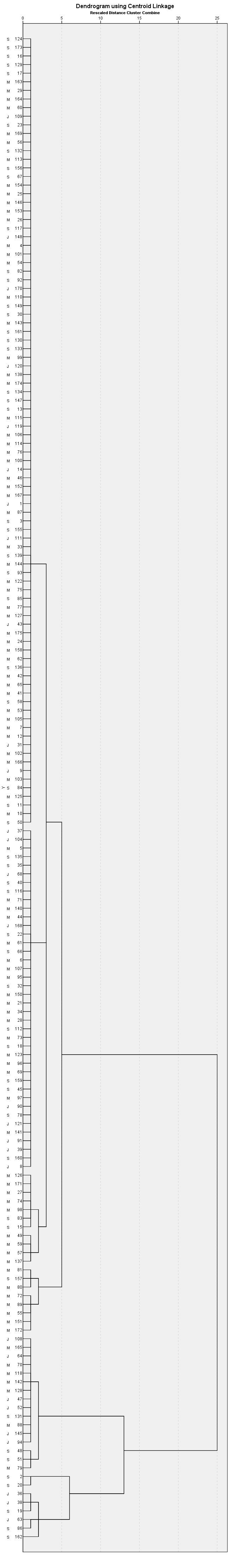

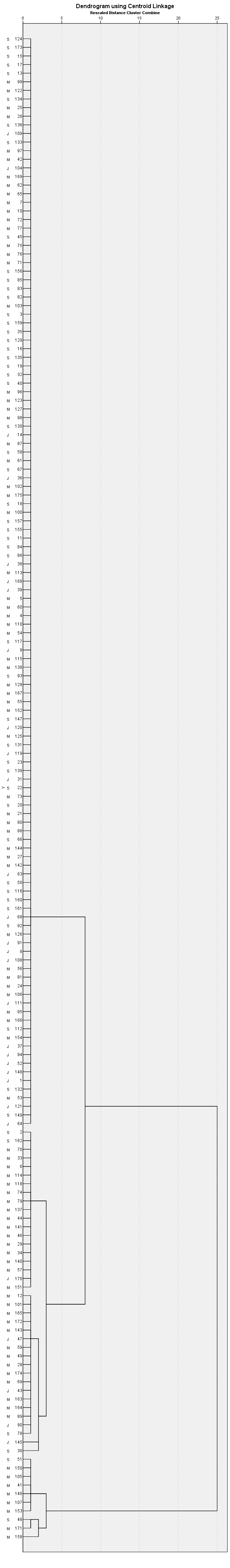

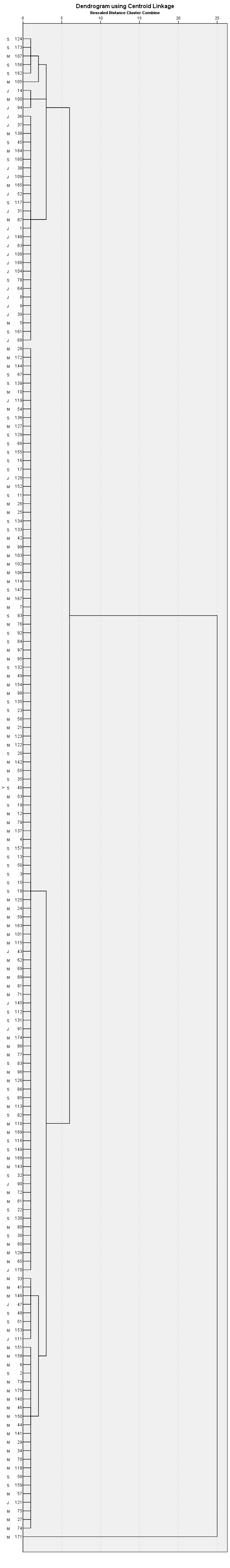

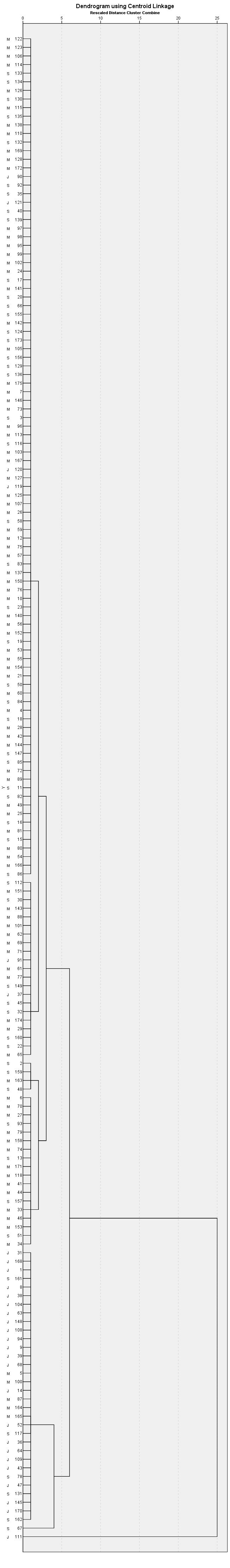


Fig S19 Fig S20 Fig S21 Fig S22 Fig. 23

Fig 19. Dendrogram plot showing Hierarchical Cluster Analysis using agglomeration schedule and Centroid clustering using Squared Euclidean Distance Interval with variables Total Hardness (TH), Calcium Hardness (CH), Total alkalinity (TA), Calcium ion (Ca^2+^) and Magnesium ion (Mg^2+^) and Label Case by District.

Fig 20. Dendrogram plot showing Hierarchical Cluster Analysis using agglomeration schedule and Centroid clustering using Squared Euclidean Distance Interval with variables Iron (Fe), Manganese (Mn) and Arsenic (As) and Label Case by District.

Fig 21. Dendrogram plot showing Hierarchical Cluster Analysis using agglomeration schedule and Centroid clustering using Squared Euclidean Distance Interval with variables Fluoride (F^-^), Ammonia (NH_3_), Nitrate (NO_3_^-^) and Chloride (Cl^-^) and Label Case by District.

Fig 22. Dendrogram plot showing Hierarchical Cluster Analysis using agglomeration schedule and Centroid clustering using Squared Euclidean Distance Interval with variables pH, Electrical Conductivity (EC), Turbidity (Tur), Color and Total dissolved solutes (TDS) and Label Case by District.

Fig 23. Dendrogram plot showing Hierarchical Cluster Analysis using agglomeration schedule and Centroid clustering using Squared Euclidean Distance Interval with all the 18 parameters as variables and Label Case by District.
